# Hypoxia-activated prodrug enhances therapeutic effect of sunitinib in melanoma

**DOI:** 10.18632/oncotarget.22944

**Published:** 2017-12-05

**Authors:** Shujing Liu, Michael T. Tetzlaff, Tao Wang, Xiang Chen, Ruifeng Yang, Suresh M. Kumar, Adina Vultur, Pengxiang Li, James S. Martin, Meenhard Herlyn, Ravi Amaravadi, Bin Li, Xiaowei Xu

**Affiliations:** ^1^ Department of Dermatology, Yueyang Hospital, Shanghai 200437, China; ^2^ Department of Pathology and Laboratory Medicine, University of Pennsylvania, Philadelphia, PA 19104, USA; ^3^ Department of Pathology, University of Texas MD Anderson Cancer Center, Houston, TX 77030, USA; ^4^ Office of Biotechnology Products, Center for Drug Evaluation and Research, U.S. Food and Drug Administration, Silver Spring, MD 20993, USA; ^5^ Department of Dermatology, Xiangya Hospital, Central South University, Changsha 410008, China; ^6^ The Wistar Institute, Philadelphia, PA 19104, USA; ^7^ Leonard Davis Institute of Health Economics, Philadelphia, PA 19104, USA; ^8^ Department of Medicine, University of Pennsylvania, Philadelphia, PA 19104, USA

**Keywords:** TH302, sunitinib, hypoxia, melanoma, treatment

## Abstract

Angiogenesis is a critical step during tumor progression. Anti-angiogenic therapy has only provided modest benefits in delaying tumor progression despite its early promise in cancer treatment. It has been postulated that anti-angiogenic therapy may promote the emergence of a more aggressive cancer cell phenotype by generating increased tumor hypoxia—a well-recognized promoter of tumor progression. TH-302 is a 2-nitroimidazole triggered hypoxia-activated prodrug (HAP) which has been shown to selectively target the hypoxic tumor compartment and reduce tumor volume. Here, we show that melanoma cells grown under hypoxic conditions exhibit increased resistance to a wide variety of therapeutic agents *in vitro* and generate larger and more aggressive tumors *in vivo* than melanoma cells grown under normoxic conditions. However, hypoxic melanoma cells exhibit a pronounced sensitivity to TH-302 which is further enhanced by the addition of sunitinib. Short term sunitinib treatment fails to prolong the survival of melanoma bearing genetically engineered mice (*Tyr::CreER; BRaf^CA^;Pten^lox/lox^*) but increases tumor hypoxia. Long term TH-302 alone modestly prolongs the overall survival of melanoma bearing mice. Combination therapy of TH-302 with sunitinib further increases the survival of treated mice. These studies provide a translational rationale for combining hypoxic tumor cell targeted therapies with anti-angiogenics for treatment of melanoma.

## INTRODUCTION

Melanoma is the deadliest form of skin cancer. The incidence of melanoma in the population is steadily rising [1, 2]. Long term survival of melanoma patients is still poor despite recent progresses in targeted therapies and immunotherapies [3, 4]. A key contributor to the poor prognosis of advanced stage melanoma is that a majority of melanomas are refractory or quickly become resistant to systemic therapies [5]. Therefore there is an urgent need to identify new therapeutic agents and/or novel treatment strategies to improve outcomes in patients with advanced stage melanoma.

A tumor relies on a steadily increasing vascular supply to sustain its progressive growth. Anti-angiogenic therapy is predicated on the concept that blocking the formation of new blood vessels will limit the capacity of a tumor to increase its volume [6]. A number of molecules targeting the vascular endothelial growth factor (VEGF)-dependent pro-angiogenic signaling pathways are in various stages of development or clinical application [7]. However, the currently available VEGF pathway inhibitors have significant limitations which include limited benefits in overall survival and the rapid development of treatment resistance. In addition, anti-angiogenic agents such as sunitinib (a receptor tyrosine kinase inhibitor) and VEGF inhibitors may paradoxically enhance tumor progression by generating a more aggressive tumor phenotype [7–9]. Among the observed mechanisms demonstrated to abrogate sensitivity to anti-angiogenic therapy is the observation that VEGF inhibitor therapy induces hypoxia dependent alterations in gene expression resulting in an *increase* in the invasive and/or metastatic capabilities of different tumor types [7, 10–12]. We and others previously showed that melanoma cells exposed to hypoxic growth conditions show upregulation of Snail1, Oct4 and Wnt5a expression [13–15]. All of these events occur in a hypoxia inducible factor (HIF)-dependent fashion. The expression of these genes correlates with the development of increased invasive and migratory capabilities and resistance to targeted therapy by melanoma cells [15–19]. These findings argue that hypoxia triggers an adaptive response by tumor cells that culminates in their capacity to survive and find more favorable (i.e. normoxic) growth conditions. These observations underscore the need for targeting hypoxic cancer cells in solid tumors in order to achieve better treatment efficacy.

TH-302 is a 2-nitroimidazole triggered hypoxia-activated prodrug (HAP). Under hypoxic conditions, TH-302 releases bromo-isophosphoramide mustard (Br-IPM), which induces DNA crosslinking [20]. Pre-clinical models demonstrate that TH-302 exhibits a broad spectrum of hypoxia-dependent cytotoxicity towards many different human cancer cell lines [21]. Early phase clinical trials showed safety and tolerance of TH-302 in patients with different human cancers. One TH-302 early phase trial demonstrated a partial response in a patient with metastatic melanoma [22, 23].

Here, we show that hypoxic melanoma cells were more aggressive *in vivo*. TH-302 enhanced the inhibitory effect of sunitinib on melanoma cells in 3D culture. Short-term treatment of sunitinib did not show any survival benefit but increased tumor hypoxia in *Tyr::CreER; BRaf^CA^; Pten^lox/lox^* mice. Long-term TH-302 treatment alone moderately prolonged the overall survival of melanoma bearing mice. In contrast, the combination of TH-302 and sunitinib significantly inhibited melanoma growth in both chemotherapeutic and chemoprevention regimens.

## RESULTS

### Hypoxia promotes melanoma aggressive behavior *in vivo*

**Figure 1:**
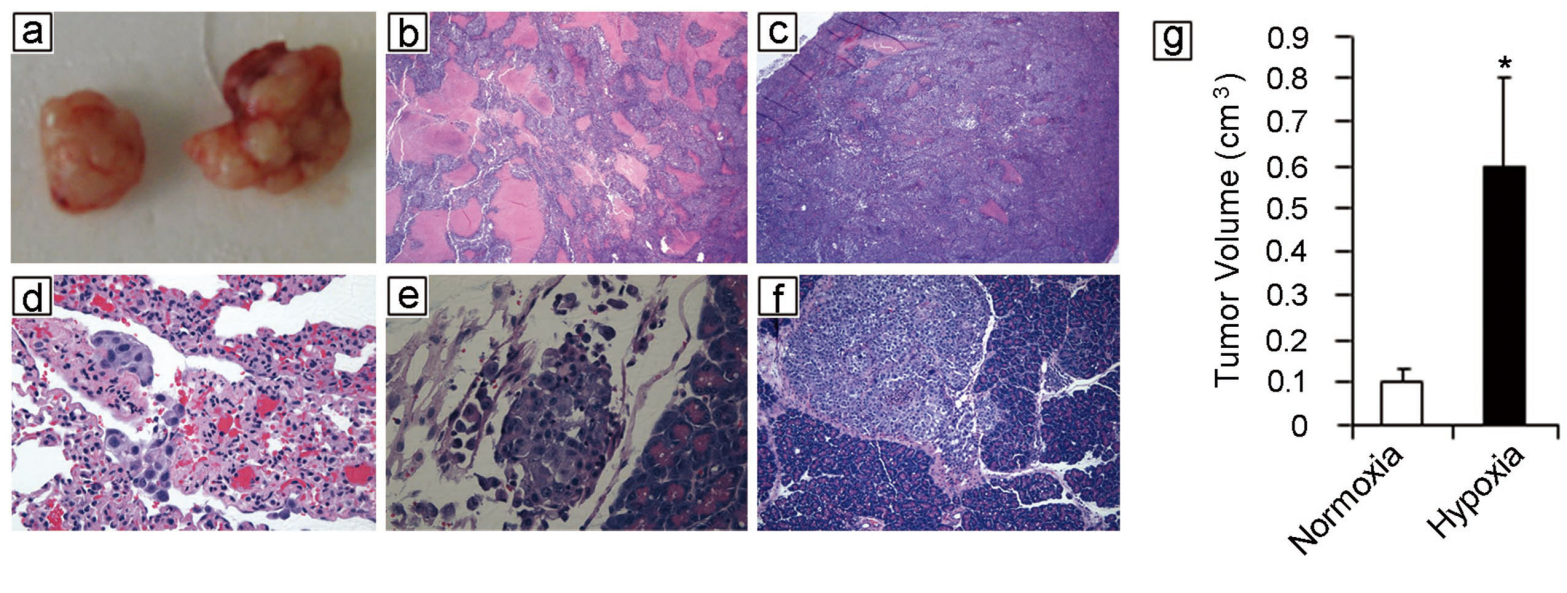
Hypoxia potentiates melanoma growth and metastasis *in vivo* Human WM115F melanoma cells were exposed to 1% O_2_ for 72 hours. 100 viable cells were injected subcutaneously into the flanks of NOD/SCID mice (n = 4). WM115F cells grown under normoxic conditions were used as control. The mice were followed for 45 days and then sacrificed. All mice that received hypoxia treated melanoma cell developed tumors, while 3 of 4 mice that received 100 normoxic cells developed tumors. **(a)** Left side: representative xenograft formed by 100 melanoma cells cultured under normoxic conditions. Right side: representative xenograft formed by 100 melanoma cells cultured under hypoxia. **(b)** 10^6^ of hypoxia-treated melanoma cells were injected subcutaneously, the same number of melanoma cells grown under normoxia were used as controls. H&E staining of a representative xenograft formed by normoxia-treated melanoma cells reveals extensive necrosis in the tumor (Arrow points to areas with tumor necrosis) (25x). **(c)** H&E staining of a xenograft tumor formed by hypoxia-treated melanoma cells reveals significantly less tumor necrosis. **(d-f)** Histology of spontaneous metastases developed by hypoxic melanoma cells: lung metastasis (d, 400x); lymphovascular invasion (e, 400x); pancreas metastasis (f, 100x). **(g)** Tumor volume. Hypoxia-treated melanoma cells form significantly larger tumors than melanoma cells grown under normoxic conditions. ^*^ indicates p<0.01.

To assess the effects of hypoxia on melanoma cells, WM115F melanoma cells (derived from a primary vertical growth phase melanoma) were exposed to hypoxia (1% O_2_) for 72 hours. Viable WM115F tumor cells were counted and used for the following experiments, and viable WM115F cells grown under normoxic conditions were used as controls. To assess tumorigenicity, only 100 hypoxia or normoxia-treated WM115F melanoma cells were injected subcutaneously into the flanks of NOD/SCID mice. The mice were sacrificed 45 days later and tumor size was measured. 4 of 4 mice receiving hypoxia-treated tumor cells developed palpable tumors, whereas 3 of 4 mice that received normoxia-treated tumor cells developed palpable tumors. The tumors that developed from hypoxia-treated cells were significantly larger than these from normoxia-treated tumor cells (Figure 1a, 1g), suggesting that melanoma cells adapted to hypoxic growth conditions survive better *in vivo*. To assess tumor proliferation and metastasis, 10^6^ hypoxic or normoxic melanoma cells were injected subcutaneously into NOD/SCID mice. Tumor size was measured. All the mice developed tumor nodules, and they were sacrificed 56 days after inoculation of the tumor. Subcutaneous tumor sizes were similar in the two groups (data not shown). However, two of four mice that received hypoxia-treated WM115F cells developed ascites, whereas none of the mice that received normoxia-treated cells developed ascites. Necropsy was performed and mouse organs were harvested for histological analysis. The mice that developed ascites had numerous small tumor deposits scattered throughout the abdominal cavity, whereas the tumors in mice that received normoxia-treated cells were all confined to the subcutaneous injection site. In addition, xenografts formed by normoxia-treated cells contained areas of geographic necrosis (Figure 1b; expansile pink areas), whereas xenografts formed by hypoxia-treated cells had significantly less tumor necrosis (Figure 1c). In addition, mice receiving hypoxia-treated melanoma cells developed metastatic tumor deposits in the lung (Figure 1d), lymphatics (Figure 1e) and pancreas (Figure 1f), whereas none of the mice receiving normoxia-treated cells developed metastases. Together, these data demonstrate that tumor cells subjected to hypoxia exhibit more aggressive phenotypes *in vivo*.

### Hypoxia promotes melanoma resistance to targeted therapeutic agents

**Figure 2:**
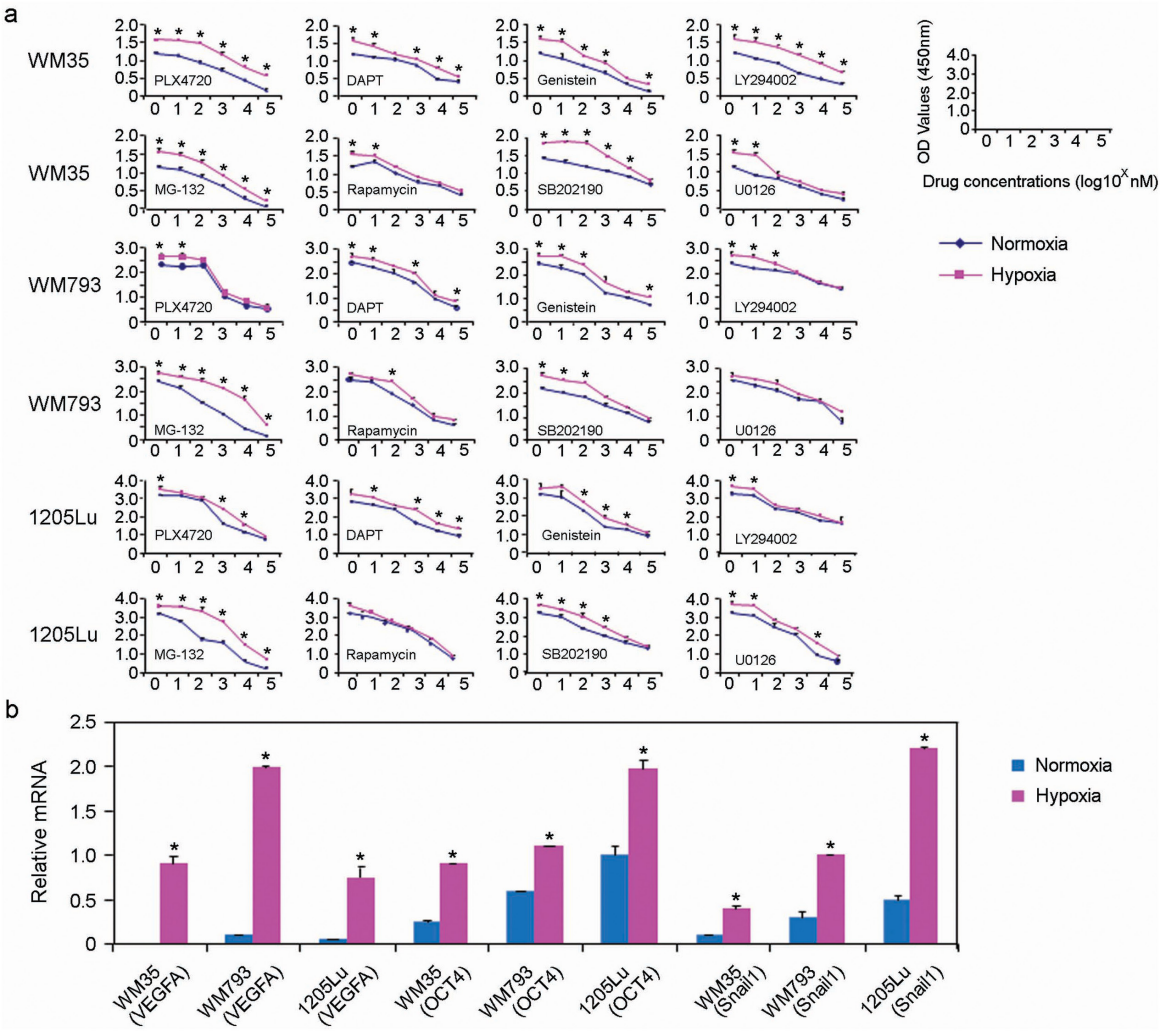
Hypoxia increases melanoma cell resistance to targeted therapeutic agents **(a)** WST-1 cell proliferation assays were used to assess cell proliferation in response to chemotherapeutic agents, including PLX-4720 (B-Raf inhibitor), DAPT (Notch inhibitor), Genistein (protein-tyrosine kinase inhibitor), and Ly294002 (PI3-K inhibitor), PLX-4720 (B-Raf inhibitor), DAPT (Notch inhibitor), Genistein (protein-tyrosine kinase inhibitor), Ly294002 (PI3-Kinase inhibitor), MG-132 (Proteasome inhibitor), Rapamycin (mTOR inhibitors), SB2012190 (MAP kinase inhibitor) and U0126 (MEK inhibitor). Hypoxia (red lines) or normoxia-treated (blue lines) melanoma cells were incubated with these agents for 24 hours (n = 3 replicate experiments for each cell line with each drug; ^*^ indicates p< 0.05 comparing hypoxia-treated to normoxia-treated cells). **(b)** Relative mRNA expression of *VEGFA, Oct4*, and *Snail* in the indicated cell lines grown under normoxic conditions (blue boxes) versus hypoxic conditions (red boxes).

Next, we assessed the susceptibility of melanoma cells grown in culture under hypoxic conditions to various targeted therapeutic agents, including PLX-4720 (B-Raf inhibitor), DAPT (Notch inhibitor), Genistein (protein-tyrosine kinase inhibitor), Ly294002 (PI3-Kinase inhibitor), MG-132 (Proteasome inhibitor), Rapamycin (mTOR inhibitors), SB2012190 (MAP kinase inhibitor), and U0126 (MEK inhibitor). As shown in Figure 2a, WM35 (derived from a radial growth phase-only primary melanoma), WM793 (derived from a vertical growth phase primary melanoma) and 1205Lu melanoma cells (derived from a melanoma metastasis) grown under hypoxic conditions exhibited a statistically significant reduction in their susceptibility to various targeted therapeutic agents compared to control cells grown under normoxic conditions. Similar results were observed when these cells were treated with MG-132 (Proteasome inhibitor), Rapamycin (mTOR inhibitors), SB2012190 (MAP kinase inhibitor), and U0126 (MEK inhibitor). We then examined the expression of genes known to mediate the cellular response to hypoxia, particularly factors known to play a role in melanoma cell invasiveness and motility [24–26]. There was a statistically significant increase in the expression of VEGFA, OCT4 and Snail1 when the cells were grown under hypoxic conditions compared to cells grown under normoxic conditions in all three melanoma cell lines tested (Figure 2b).

### TH-302 increases the inhibitory effect of sunitinib *in vitro*

**Figure 3:**
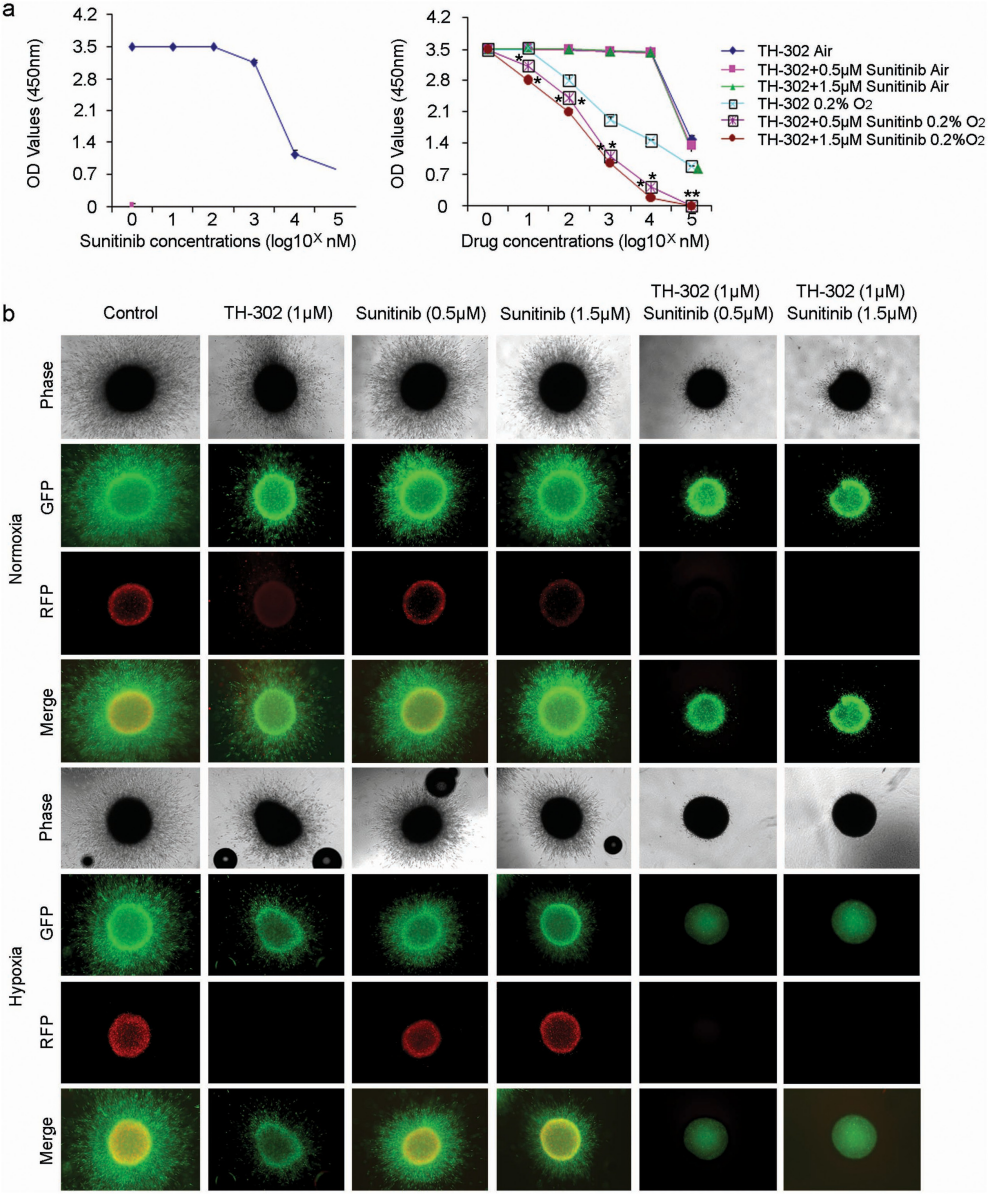
TH-302 increases the inhibitory effect of sunitinib *in vitro* **(a)** Left panel: Sunitinib alone has little effect on the growth of 1205Lu melanoma cells at concentrations less than 500 nM. Cell survival was assessed by WST-1 assay. Right panel: Effects of increasing concentrations of TH-302 together with variable concentrations of sunitinib (0, 0.5μM, 1.5μM) on 1205Lu melanoma cells grown in normoxic and hypoxic conditions. Cell survival was assessed by WST-1 assay. (n = 3 replicate experiments; ^*^indicates p<0.05 compared with control). **(b)** Effects of sunitinib, TH-302 or sunitinib plus TH-302 on 1205Lu cells grown in 3D culture. 1205Lu cells were cultured as spheroids in collagen gel and treated with the indicated concentrations of TH-302, sunitinib or TH-302 plus sunitinib for 72 hours under normoxia or hypoxia. Spheroids were then stained with calcein-AM and imaged with a confocal microscope. There was complete inhibition of melanoma growth with combination of TH-302 and sunitinib under hypoxic conditions.

Sunitinib inhibits cellular signaling by targeting multiple receptor tyrosine kinases (RTKs), including the kinases for platelet-derived growth factor (PDGFR) and vascular endothelial growth factor (VEGFR). Suntinib also reduces tumor vascularization and triggers cancer cell apoptosis. We first tested the effect of sunitinib and TH-302 in conventional 2D culture assays. Sunitinib did not significantly affect cell survival at concentrations less than ∼0.5μM under hypoxic growth conditions (Figure 3a, left panel). 0.5 and 1.5 μM of sunitinib was used in the additional experiments. Under normoxic conditions, TH-302 had relatively little impact on 1205Lu cell survival (Figure 3a, right panel). However, TH-302 significantly inhibited 1205Lu cell survival under hypoxic culture conditions. This effect was significantly amplified by the addition of sunitinib (Figure 3a, right graph). Nearly identical results were observed for WM35 and WM793 melanoma cell lines (data not shown).

We then tested the effects of TH-302 and sunitinib under three-dimensional growth conditions. We generated 1205Lu spheroids and implanted these spheroids into a collagen gel where they were subjected to varying concentrations of TH-302 and sunitinib and cultured under either normoxic or hypoxic conditions. The core of tumor spheroids are known to be hypoxic even when the spheroids are cultured under normoxic conditions [27]. After 72 hours of exposure, the cells were treated with calcein AM, which stains living cells green. Under normoxic conditions, there was only a mild inhibitory effect of either TH-302 or increasing concentrations of sunitinib on 1205Lu melanoma spheroid growth and infiltration of the surrounding collagen gel. However, there was significant inhibition of 1205Lu melanoma spheroid growth in the presence of both TH-302 and sunitinib (Figure 3b, upper panel). This effect was further amplified under hypoxic growth conditions, where there was virtually complete inhibition of 1205Lu melanoma spheroid growth in the presence of both TH-302 and sunitinib. In contrast, the effect of either was mild when they were used individually under hypoxic conditions (Figure 3b, lower panel). Together, these findings suggest a synergistic effect of TH-302 and sunitinib on the growth of melanoma cells in 3D culture.

### Hypoxia is present in mouse melanoma *in vivo*

**Figure 4:**
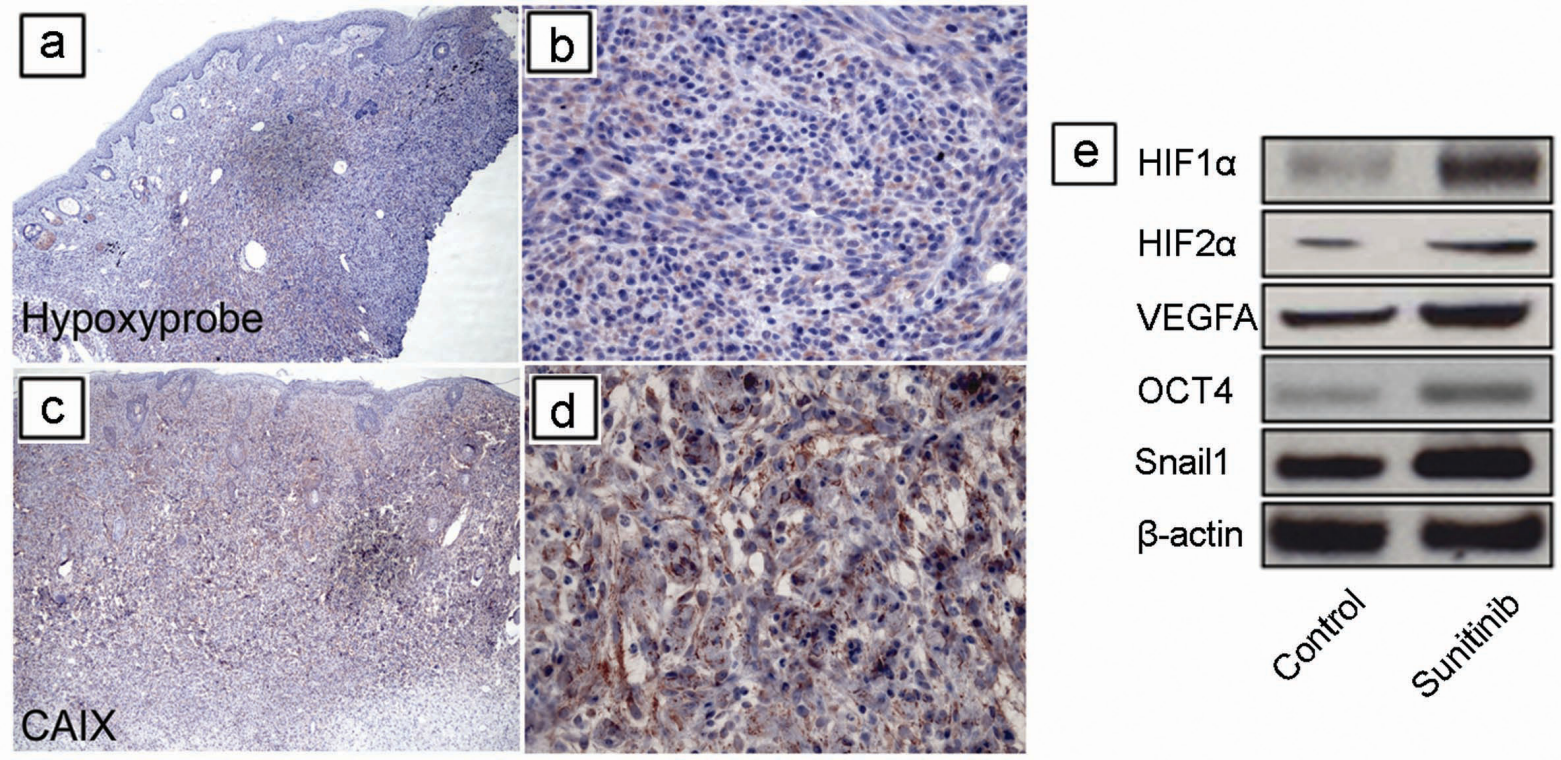
Short-term sunitinib therapy increases tumor hypoxia and HIF regulated proteins *in vivo* Using *Tyr::CreER; Braf^CA/+^; Pten^lox/lox^* mice, melanomas were induced by applying 4-hydroxytamoxifen (4-HT) on skin for 3 days. Tumors were allowed to grow for 4 weeks until they were palpable. Melanoma bearing mice were injected with Hypoxyprobe one hour before sacrificing, and melanoma tissue was stained with an anti-pimonidazole antibody, which identified areas of hypoxia. **(a, b)** Many melanoma cells in the dermis were variably positive for Hypoxyprobe staining. **(c, d)** Tumor cells were positive for the well-known marker for hypoxia, carbonic anhydrase IX (CAIX). **(e)** Melanoma was induced in *Tyr::CreER; Braf^CA/+^; Pten^lox/lox^* mice. Five mice were used in each group. Tumors were allowed to grow for 3 weeks until palpable tumors were evident. The mice were then treated for one week (short term) with vehicle (DMSO) or sunitinib (See Supplementary Figure 1 for scheme). Expression of HIF-1α, HIF-2α, VEGF-A, Snail1, Oct4 proteins was determined by western blot analysis in melanomas after treatment (n = 3 replicate experiments). β-actin was used as a loading control.

To study the effects of TH-302 and sunitinib *in vivo*, we utilized a genetically engineered mouse (GEM) model for melanoma [28]. First, we addressed whether melanomas arising in this model develop hypoxia as in human melanoma [13–15]. Using *Tyr::CreER; Braf^CA^; Pten^lox/lox^* mice, melanomas were generated by applying 4-hydroxytamoxifen (4-HT) on the skin for 3 days. The resultant tumors grew at the area of 4-HT induction as previously described [29]. To assess the presence of tumor hypoxia, tumors were allowed to grow for 4 weeks until they were palpable. Melanoma bearing mice were injected with hypoxyprobe one hour before sacrifice. Harvested melanoma tissue was stained with an anti-pimonidazole antibody, which identified areas with hypoxia. Many melanoma cells in the dermis were positive for hypoxyprobe staining (Figure 4a and 4b). To further confirm the findings, we also performed immunohistochemical staining using a monoclonal antibody against the hypoxia marker carbonic anhydrase IX (CAIX). CAIX immunostaining showed similar changes to those seen by hypoxyprobe staining (Figure 4c and 4d). Together, these data demonstrate that hypoxia is present in melanoma tissue in this model.

### Short term sunitinib and TH-302 treatment of established melanoma *in vivo*

Previous studies in xenograft models showed that short term sunitinib treatment paradoxically increased tumor growth and tumor hypoxia in xenograft models [12, 30]. To study whether short-term sunitinib increases tumor hypoxia in the GEM melanoma model, melanomas were induced and allowed to grow for 3 weeks until they were palpable. These mice were then treated for one week with either vehicle control or sunitinib (Supplementary Figure 1) after which they were sacrificed. Tumor tissues were then snap frozen for further analysis. Western blot analysis of protein extracts from these melanomas demonstrated increased expression of HIF-1α, HIF-2α, VEGF-A, Snail1, and Oct4 protein in response to sunitinib treatment compared to vehicle controls (Figure 4e).

**Figure 5:**
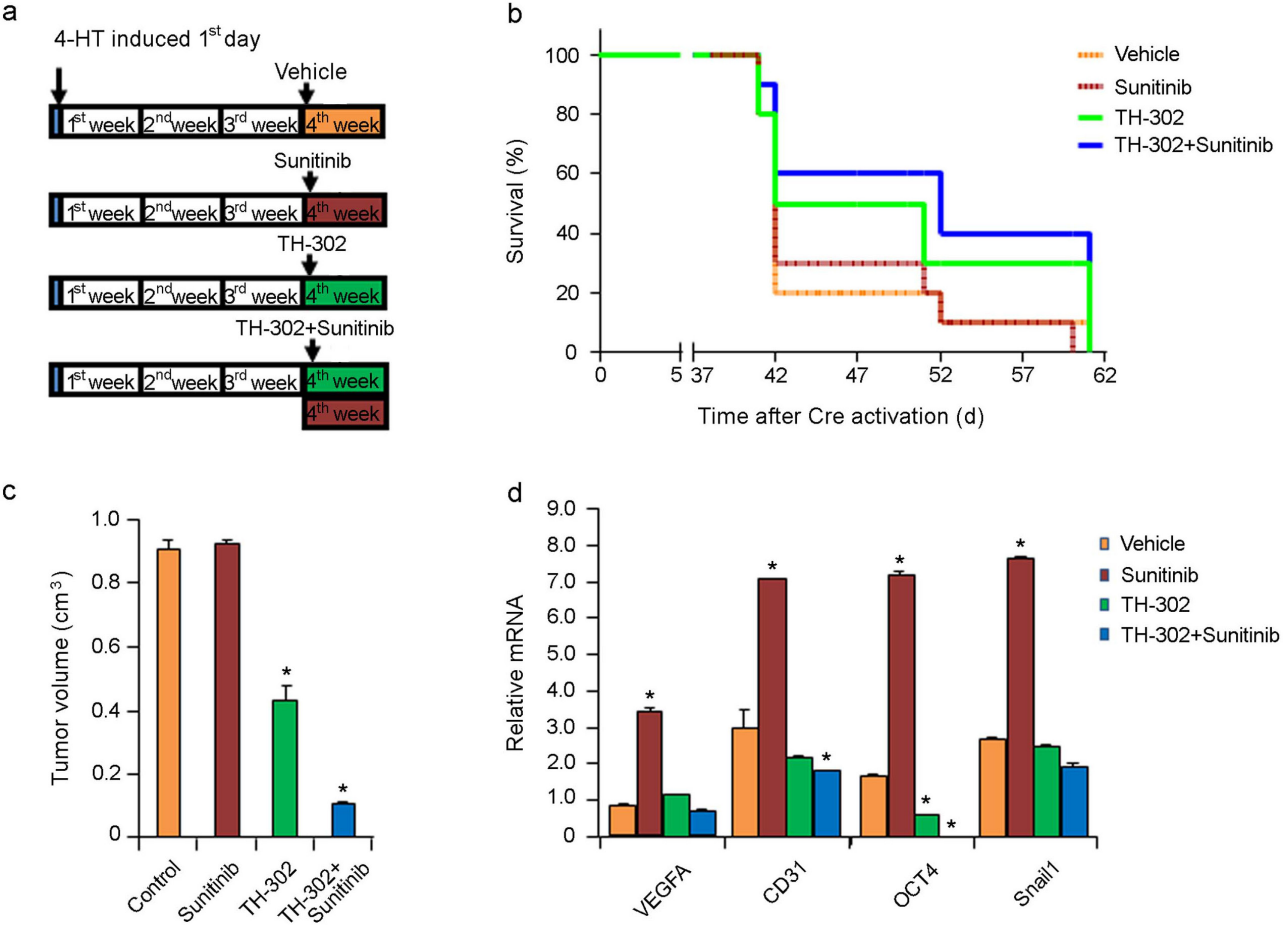
Effects of short term sunitinib and TH-302 therapy *in vivo* **(a)** Schemes of melanoma induction and treatments. Melanoma was induced by applying 4-HT on *Tyr::CreER; Braf^CA/+^; Pten^lox/lox^* mice for 3 days. Tumors were allowed to grow for 3 weeks until palpable tumors were evident. The mice were then treated for one week (short term) with vehicle control, TH-302, sunitinib, or TH-302 plus sunitinib as indicated. Twenty mice were used in each group. **(b)** Effect of short term treatments on mouse survival. Mice were euthanized according to the standard score of body condition. Kaplan-Meier survival analysis demonstrated that short-term sunitinib alone did not affect the lifespan of melanoma bearing mice compared to that of control. In contrast, short term TH-302 modestly increased mouse survival, while addition of sunitinib added little efficacy. **(c)** Effect of sunitinib and TH-302 on tumor volume. A subset of treated mice was euthanized 40 days after melanoma induction and tumor volume was measured. ^*^ indicates p<0.05. **(d)** Quantitative RT-PCR assay for *VEGF-A, CD31, Oct4* and *Snail1* mRNA expression in melanoma bearing mice treated with short term sunitinib, TH-302 or TH-302 plus sunitinib (n = 3 replicate experiments). Short-term treatment with sunitinib resulted in increased *VEGF-A, CD31, Oct4*, and *Snail1* mRNA expression in melanoma, while treatment with TH-302 or Th-302 plus sunitinib prevented their increased expression. β-actin was used as an internal control. (^*^p< 0.01 compared with control).

To study the treatment effect of short-term TH-302 or TH-302 plus sunitinib on melanoma progression, mice with established melanoma were treated with either vehicle control, sunitinib, TH-302, or a combination of sunitinib and TH-302 for one week (Figure 5a). The mice were followed daily and sacrificed once they exceeded the standard body condition score. A subset of treated mice was euthanized 40 days after melanoma induction. Sunitinib-treated melanomas showed no significant difference in tumor volume comparing to the control mice (Figure 5c). In contrast, one week treatment with TH-302 alone or in combination with sunitinib resulted in a significant reduction in tumor volume (Figure 5c) at day 40. One week sunitinib treatment also resulted in increased mRNA expression levels of *VEGFA, CD31* (an endothelial cell marker), *Oct4* and *Snail* compared to the control treatment group, supporting that short term sunitinib treatment induces greater hypoxia in the tumor tissue (Figure 5d). In contrast, addition of TH-302 to sunitinib significantly reduced the mRNA levels of *CD31* and *Oct4* expression in the tumor tissues compared to those of control treatment (Figure 5d). Treatment of TH-302 reduced *Oct4* expression compared to control.

All the control mice had to be sacrificed on day 60 due to the tumor size; treatment with one week of sunitinib alone exerted minimal effects on overall survival (Figure 5b). However, one week treatment with TH-302 and sunitinib resulted in a moderate increase in overall survival (Figure 5b) that was statistically significant (median survival 50 days; p=0.0217 log rank test compared to control group). One week treatment with single agent TH-302 (median survival 41 days; p=0.1804 compared to control group) or sunitinib (median survival 41 days; p=0.8217 compared to control group) failed to prolong the survival of treated mice.

### Long term sunitinib and TH-302 treatment of established melanoma *in vivo*

**Figure 6:**
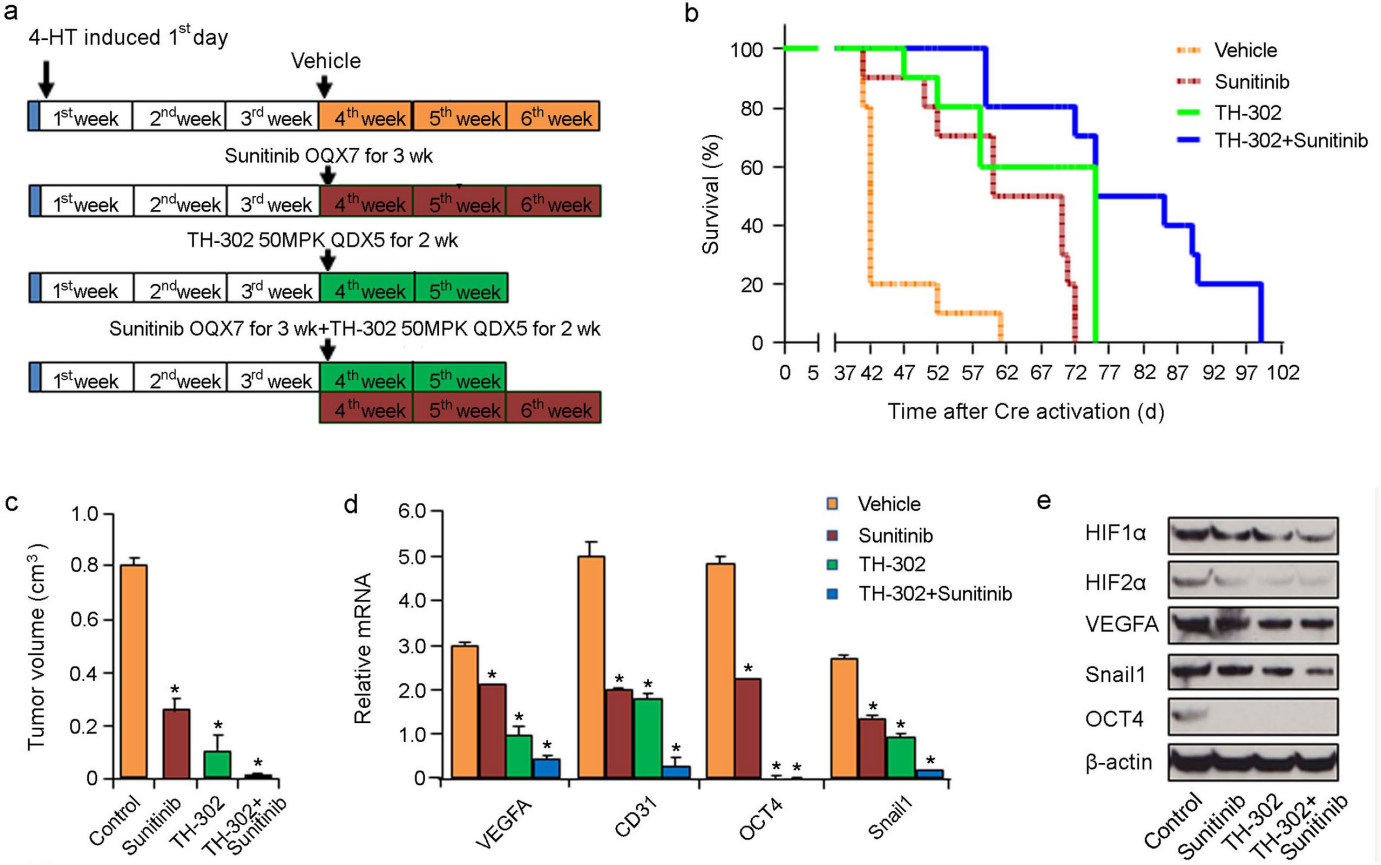
Effects of long term TH-302 and sunitinib therapy *in vivo* **(a)** Scheme of experiments. Melanoma was induced by applying 4-HT on *Tyr::CreER; Braf^CA/+^; Pten^lox/lox^* mice. Twelve mice were used in each group. Tumors were allowed to grow for 3 weeks until palpable tumors were evident. The mice were then treated with vehicle (DMSO), TH-302, sunitinib, or TH-302 plus sunitinib for the indicated time periods. **(b)** Kaplan-Meier survival analysis demonstrated that TH-302 plus sunitinib (long-term) therapy significantly extended the lifespan of melanoma bearing mice compared with that of long-term TH-302 or sunitinib treatment. Mice were euthanized according to the standard body condition score. **(c)** Effect of long term therapy on tumor volume. A subset of treated mice were euthanized 40 days after melanoma induction and tumor volume was measured. ^*^ indicates p<0.05. **(d)** Quantitative RT-PCR assay of *VEGF-A, CD31, Snail1* and *Oct4* mRNA expression in sunitinib, TH-302, or TH-302 plus sunitinib long-term treatment mouse groups (n = 3 replicate experiments; ^*^indicates p< 0.01 compared with vehicle control). β-actin is used as an internal control. **(e)** Expression of HIF-1α, HIF-2α, VEGF-A, Snail1 and Oct4 proteins was determined by western blot analysis in melanoma bearing mice treated with long term sunitinib, TH-302 or TH-302 plus sunitinib. β-actin was used as a loading control.

We next assessed whether long term therapy with sunitinib and/or TH-302 could impact the overall survival of melanoma bearing mice. Melanomas were induced and allowed to grow for 3 weeks until they were palpable. These mice were then treated with either vehicle control, sunitinib, TH-302 or sunitinib plus TH-302 (Figure 6a for details of respective treatment regimens). Both long term sunitinib treatment (median survival 70 days; p<0.001 log rank test compared to vehicle control group) or TH-302 treatment (median survival 74 days; p<0.001 log rank test compared to vehicle control group) significantly increased mouse survival compared to vehicle alone (Figure 6b). The combination of sunitinib and TH-302 resulted in a further increase in overall melanoma survival (median survival 84 days) compared to either sunitinib or TH-302 alone (Figure 6b). A subset of treated mice was euthanized 40 days after melanoma induction while they were still on treatment. Sunitinib, TH-302, and TH-302 plus sunitinib significantly decreased tumor volume on day 40 (Figure 6c). We measured HIF- and VEGF-target expression in the tumor tissues. Three weeks of sunitinib, TH-302, or combination therapy induced an abrogation of the hypoxia induced mRNA and protein expression programs including both mRNA and protein levels of VEGF-A, CD31, Snail, and Oct-4; and protein levels of HIF-1α and HIF-2α, (Figure 6d and 6e).

### Effects of TH-302 and sunitinib tumor prevention regimen on melanoma *in vivo*

**Figure 7:**
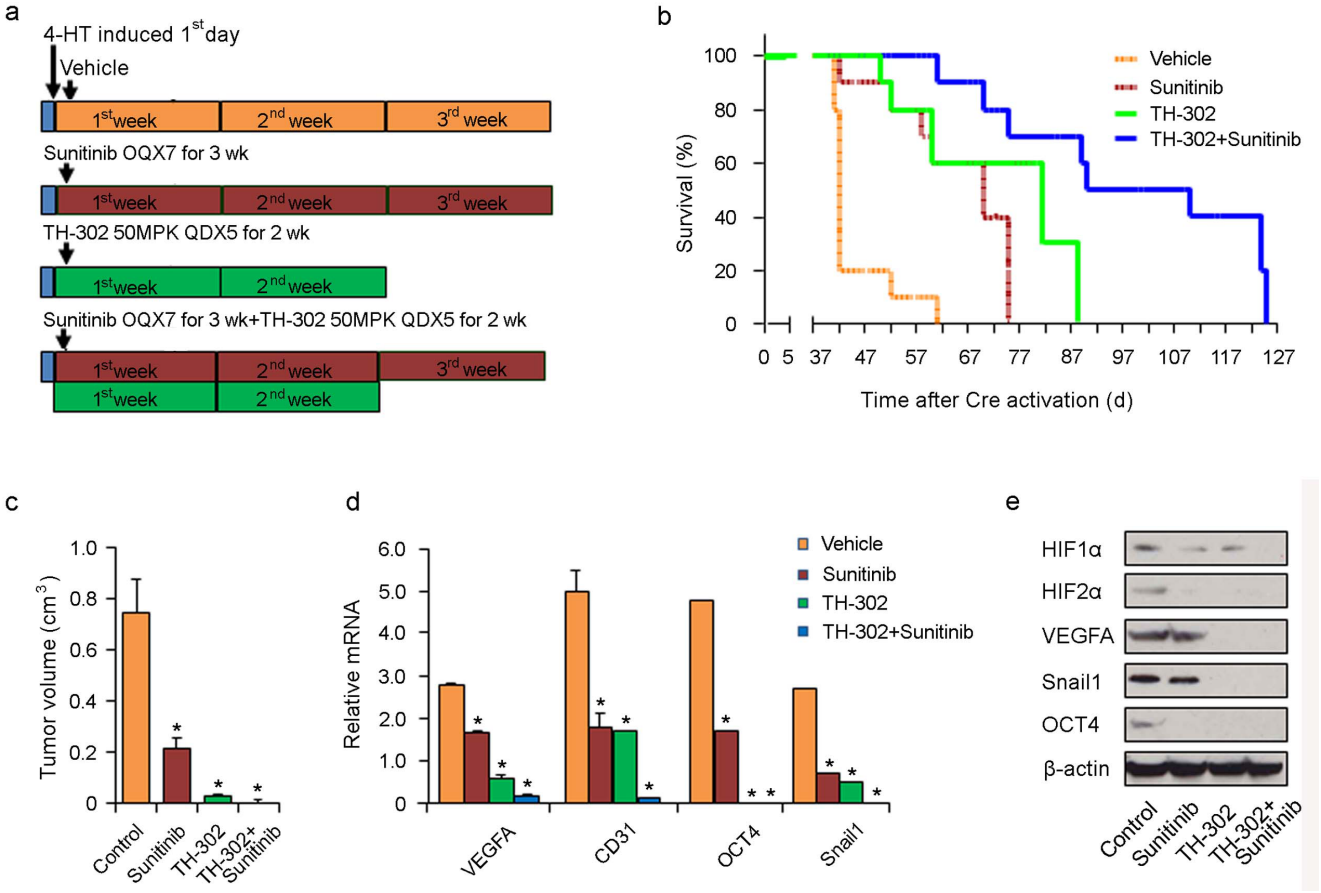
Effects of long term TH-302 and sunitinib prevention regimen *in vivo* **(a)** Scheme of experiments. Melanoma was induced by applying 4-HT for 3 days on Tyr::CreER; Braf^CA/+^; Pten^lox/lox^ mice. Twelve mice were used in each group. On the second day of induction, the mice were treated with vehicle (DMSO), sunitinib, TH-302 or sunitinib plus TH-302 for the time periods indicated. **(b)** Kaplan-Meier survival analysis demonstrated that prevention scheme with long term TH-302 plus sunitinib treatment significantly extended the lifespan of melanoma-bearing mice compared to TH-302 or sunitinib treatment. Mice were euthanized according to the standard body condition score. **(c)** Effect of therapies on tumor volume. A subset of treated mice were euthanized 40 days after melanoma induction and tumor volume was measured. ^*^ indicates p<0.05. **(d)** Quantitative RT-PCR assay of *VEGF-A, CD31, Snail1* and *Oct4* mRNA expression in tumors treated with vehicle control, sunitinib, TH-302 or TH-302 plus sunitinib (n=3 replicate experiments; ^*^indicates p< 0.01 compared with vehicle control). β-actin was used as an internal control. **(e)** Expression of HIF-1α, HIF-2α, VEGF-A, Snail1 and Oct4 proteins was determined by western blot analysis in melanoma bearing mice treated with long term sunitinib, TH-302 or TH-302 plus sunitinib. β-actin was used as a loading control.

Melanomas were generated by applying 4-HT on the skin of *Tyr::CreER; Braf^CA^; Pten^lox/lox^* mice for 3 days. Treatment was initiated after the first day of tumor induction. Treatment consisted of either vehicle control for 3 weeks, sunitinib for 3 weeks, TH-302 for 2 weeks, or their combination (Figure 7a). As shown in Figure 7b, earlier intervention (i.e. prevention regimen) with the long term combination of sunitinib and TH-302 resulted in an increased overall survival (median survival 112 days; p<0.001 log rank test) compared to vehicle control; sunitinib alone (median survival 75 days; p<0.001) or TH-302 alone (median survival 84 days; p<0.001). A subset of treated mice was euthanized 40 days after melanoma induction and the tumor volume was measured. TH-302 and TH-302 plus sunitinib significantly decreased tumor volume (Figure 7c). The improved survival seen in chemoprevention strategies coincided with statistically significant reductions in hypoxia-induced mRNA and protein expression, including HIF-1α, HIF-2α, VEGF-A, CD31, Snail, and Oct-4 (Figure 7d and 7e).

## DISCUSSION

Our study showed that melanoma cells grown under hypoxic conditions were more tumorigenic and exhibited more aggressive phenotypes *in vivo*. Short term sunitinib treatment failed to prolong survival of mice but increased tumor hypoxia. TH-302 prolonged the overall survival of melanoma bearing mice using different treatment regimens of varying duration—both alone but especially in combination with sunitinib. This effect correlated with a reduction in the expression of hypoxia-induced factors. Our data suggest that hypoxia activated prodrugs may be combined with anti-angiogenic agents to destroy hypoxic melanoma cells destined to evade antiangiogenic therapy through hypoxia-inducible genetic programs.

Sunitinib and TH-302 treatment was performed in a genetically engineered mouse (GEM) model instead of xenograft models. We reasoned that the tumor stroma and vasculature in GEM models better mimics the human tumor microenvironment than in xenograft models [31, 32]. Indeed, melanomas generated in the GEM model exhibit hypoxia, a feature commonly seen in human melanoma [33]. Our study showed that short term of sunitinib treatment enhances intratumoral hypoxia but failed to elicit any therapeutic effects, while long term treatment inhibited melanoma growth. The results are supported by prior studies by Ebos *et al*. demonstrating that sunitinib therapy exerts opposing effects on tumor growth depending on the context of tumor inoculation and the schedule of sunitinib administration in breast cancer and melanoma xenograft models [10]. Our data showed that short term sunitinib treatment is associated with more tumor hypoxia and increased expression of Snail, VEGFA, and Oct4. These genes are known to be involved in more aggressive tumor phenotypes, supporting the idea that short term angiogenesis inhibition may induce more aggressive tumor phenotypes.

It has been demonstrated that increased tumor invasiveness and/or increased lymph node and liver metastases following genetic and various pharmacologic ablations of the VEGF-receptor. These more aggressive tumor phenotypes correlated with increased tumor hypoxia. These observations directly implicate hypoxia-induced signaling cascades as the driving force behind this adaptive/evasive response to VEGF inhibition [11]. Our results further demonstrate that hypoxic growth conditions direct specific changes in melanoma cell gene expression that serve to promote enhanced tumor cell migration and tumor invasiveness. This hypoxia induced gene expression culminates in the capacity of the melanoma cell to relocate to an environment more conducive to growth. Furthermore, hypoxic tumor cells are more resistant to various therapeutic agents.

We demonstrate that administration of a single agent TH-302 results in improved melanoma survival compared to controls. TH-302 and sunitinib produce even greater improvements in overall survival compared to control and either TH-302 or sunitinib alone. The greatest benefit was observed in mice treated with long term TH-302 and sunitinib immediately after the induction of melanoma. Our study suggests that addition of a hypoxia-activated prodrug may alter the adaptive/evasive response induced by hypoxia. As has been suggested previously [10–12], our results highlight the potential benefits—if not the necessity—of enlisting additional chemotherapeutic agents together with anti-angiogenic therapy. Thus, our study provides an important translational rationale for combining TH-302 with anti-angiogenic drugs to augment the effects of anti-antiogenics in the management of melanoma and possible other cancers by specifically targeting those hypoxic cells which would otherwise emerge as a more aggressive counterpart.

Despite the recent failure of TH-302 to show significant clinical benefits in Phase III clinical trials of sarcoma and pancreatic cancer, future clinical trials combining hypoxic tumor cell targeted agents with anti-angiogenic agents, such as TH-302 in combination with sunitinib or other VEGF inhibitors, may be efficacious to combat cancer and improve patient survival.

## MATERIALS AND METHODS

### Reagents and cell culture

Mouse monoclonal HIF-1α antibody was purchased from NeoMarkers (Fremont, CA, USA); mouse monoclonal HIF-2α antibody was purchased from NOVUS Biologicals (Littleton, CO, USA); rabbit polyclonal VEGF147 antibody was purchased from Santa Cruz Biotechnology (Santa Cruz, CA, USA); rabbit monoclonal Snail1 antibody was purchased from Cell Signaling Technology (Danvers, MA, USA); rabbit polyclonal Oct4 antibody was purchased from Abcam (Cambridge, MA, USA); rabbit polyclonal CAIX antibody was purchased from NOVUS Biologicals (Littleton, CO, USA); and mouse monoclonal anti-β-actin was purchased from Sigma (Northbrook, IL, USA). Temozolomide (temodal) was purchased from TOCRIS Biosciences (Bristol, UK); PLX-4720 (B-Raf inhibitor) was purchased from Selleck (Houston, TX, USA); DAPT (Notch inhibitor) was purchased from Alexis Corporation (San Diego, CA, USA); Genistein (protein-tyrosine kinase inhibitor) was purchased from Calbiochem (La Jolla, CA, USA); LY294002 (PI3-K inhibitor) was purchased from Cell Signaling Technology (Danvers, MA, USA); MG-132 (Proteasome inhibitor) and rapamycin (mTOR inhibitor) were both purchased from Calbiochem (La Jolla, CA, USA); SB2012190 (MAP kinase inhibitor), U0126 (MEK inhibitor), and 4-hydroxytamoxifen (4-HT) were all purchased from Sigma (Northbrook, IL, USA). WM35, WM793 and 1205LU were cultured in a MCDB153/L15 medium (v/v: 4/1) supplemented with 2% FBS, insulin (5 mg/ml), 2 Mm CaCl_2_ and 100 U/ml penicillin and 100 mg/ml streptomycin (2% tumor medium). 115F cells were cultured in stem cell medium (80% DMEM/F12, 20% Knockout serum replacement, 20 ng/mLbFGF). Cells were cultured in a 5% CO_2_ incubator at 37°C.

### Generation of hypoxia tumor xenografts

All animal protocols were approved by the IACUC committee at the University of Pennsylvania. To establish hypoxic conditions, 115F cells were incubated in 1% O_2_ at 37°C for 16 hours and then harvested for injection. Non-obese diabetic (NOD)-severe combined immunodeficiency (SCID) mice were purchased from the Jackson Laboratory (Bar Harbor, ME, USA). 2 × 10^6^ normoxic 115F or hypoxic 115F cells in 100 ul Matrigel (BD Biosciences, San Jose, CA) were injected subcutaneously into the the flanks of non-obese diabetic (NOD)-severe combined immunodeficiency (SCID) mice (8 mice). Caliper measurements of the tumors were obtained every 72 hours. Tumor volume was monitored closely for 4 weeks, at which time all of the animals were sacrificed and necropsies were performed. All organs were preserved and then examined for metastasis. Tumors were excised and processed for routine histologic examination.

### Immunohistochemistry and western blot assay

Immunohistochemistry and Western blots were performed as previously described [34].

### Hypoxia treatment of melanoma cells and drug resistance studies

WM35, WM793 and 1205LU cells were seeded and incubated at 37°C in a 2% CO2 incubator for 24 hours. The media was then replaced with fresh 2% MCDB media and cultures were incubated in 1% O_2_ conditions at 37°C for 16 hours.

The hypoxic melanoma cells were washed with PBS and 1 × 10^5^ cells were plated in 6 well plates. The plates were incubated at 37°C for 24 hours and cultured with serum free MCDB medium for another 24 hours in a humidified CO_2_ incubator. The culture medium was aspirated and 2% MCDB tumor media containing different concentrations of PLX-4720, DAPT, Genistein, LY294002, MG-132, Rapamycin, SB2012190, and U0126 (with 0, 1 nM, 10 nM, 100 nM, 1μM and 10μM) was added to each well. Drug treated and control cells were incubated another 48 hours at 37°C in a humidified CO_2_ incubator before performing the WST-1 cell proliferation assay. The WST-1 cell proliferation assay was performed as described [16]. Experiments were carried out in triplicate.

### Isolation of RNA and quantitative real time PCR

Isolation of RNA and quantitative real time PCR were performed as previously described [16]. The following primers were used:

**Table:** Real time PCR primers A

Human Gene	Forward Primer	Reverse Primer
*VEGFA*	CTA CCT CCA CCA TGC CAA GT	GCA GTA GCT GCG CTG ATA GA
*Oct4*	GGC GTT CTC TTT GCA AAG GTG TTC	CTC GAA CCA CAT CCT TCT CT
*Snail1*	GAC TAG AGT CTG AGA TGC CC	CAG ACA TTG TTA AAT TGG CCG
*β-actin*	TGA CTG ACT ACC TCA TGA AGA TCC	GCC ATC TCT TGC TCG AAG TCC

**Table:** Real time PCR primers B

Mouse Gene	Forward Primer	Reverse Primer
*VEGFA*	CTG TGC AGG CTG CTG TAA CG	GTT CCC GAA ACC CTG AGG AG
*CD31*	GAC ACT ACA CCT GCA AAG TG	GCA CCG AAG TAC CAT TTC AC
*Snail1*	TTG TAA CAA GGA GTA CCT CAG	GCA GCC AGA CTC TTG GTG CTT
*OCT4*	CTG TAG GGA GGG CTT CGG GCA CTT	CTG AGG GCC AGG CAG GAG CAC GAG
*β-actin*	ATG AAG TGT GAC GTT GAC ATC CGT	CCT AGA AGC ATT TGC GGT GCA CGA TG

### Hypoxyprobe™ assay

A Hypoxyprobe™-1 Kit for the Detection of Tissue Hypoxia was purchased from Chemicon International, INC (Temecula, CA, USA). The hypoxyprobe assay was performed according to the manufacturer’s instructions.

### Mice and drug treatments

The *Tyr::CreER; BRaf^CA/+^; Pten^lox/lox^* transgenic mice were originally generated by Drs. Marcus Bosenberg (Yale) and Martin McMahon (UCSF). The protocol has been approved by the IACUC committee at the University of Pennsylvania. Genotyping and tumor induction were performed as described [28, 29]. Briefly, 4-hydroxytamoxifen (4-HT) was dissolved in DMSO to a final concentration of 65-130mM (70% Z-isomaer, Sigma). Adult (6-8 weeks of age) mice were treated topically with a 5mM 4-HT solution, which was applied using a small paint brush on the right flank, ear and tail for 3 days. TH-302 was dissolved in 0.9% NaCl and then injected intraperitoneally at a dose of 10 mg/kg, three times each week, for 3 weeks starting on the 2^nd^ (long-term chemoprevention treatment regimen) or on the 22^nd^ day (long-term chemotherapy treatment regimen) following application of 4-HT, or injected intraperitoneally at a dose of 10 mg/kg, three times each week, for 1 week starting 2^nd^ (short-term chemoprevention treatment regimen) or on the 22^nd^ day (short-term chemotherapy treatment regimen) following application of 4-HT. Sunitinib was dissolved in phosphate-citrate buffer (Ph=3.5) and administered orally at a dose of 40 mg/kg once daily for 3 weeks starting on the 2^nd^ (long-term chemoprevention treatment regimen) or 22^nd^ day (long-term chemotherapy treatment regimen) after application of 4-HT, or orally at a dose of 40 mg/kg once daily for 1 week starting on the 2^nd^ (short-term chemoprevention treatment regimen) or 22^nd^ day (short-term chemotherapy treatment regimen) following application of 4-HT. TH-302 plus sunitinib treatment regimens applied combinations of the strategies described above. Control animals in the melanoma studies were administered with the relevant solvent.

Tissues were prepared for analysis as previously described [35, 36]. We used 20 mice for each group experiment; these 20 mice were separated into two groups. The first group of 10 mice was sacrificed on the 40^th^ day after 4-HT tumor induction, and the volume of the induced tumors was measured. In addition, where indicated freshly isolated melanoma tissue was used for analyzing gene and protein expression. The remaining 10 mice were utilized in survival studies. The mice were euthanized when they achieved the standard body condition score.

### Sphere formation assays

Melanoma spheroids were prepared using the liquid overlay method as previously described [37]. ∼5000 1205Lu melanoma cells were seeded onto a 96-well plate coated with 1.5% agar (Difco, Sparks, MD) and incubated for 72 hours to allow for the formation of three-dimensional spheroids. Spheroids were then harvested and were implanted into a gel of bovine collagen I containing DMEM, l-glutamine, and 2% fetal bovine serum. Spheroids were then cultured under normoxic or hypoxic conditions as described above and incubated in the presence of TH-302 (1 μM), sunitinib (0.5 μM or 1.5 μM), or TH-302 plus sunitinib (0.5 μM or 1.5 μM), and left to grow for 72 hours. Spheroids were then washed twice in PBS before being treated with calcein-AM and ethidium bromide (Molecular Probes, Eugene, OR) for 1 hour at 37°C according to the manufacturer’s instructions. Photographs of spheroids were taken using a Nikon-300 inverted fluorescence microscope.

### Statistical analysis

Data are represented as mean +/− SEM. Student’s *t* test or one way analysis of variance (ANOVA) were used to analyze differences in gene expression, cell viability, and proliferation data amongst the experimental groups in various treatment protocols. A two tailed p-value<0.05 was considered statistically significant.

## SUPPLEMENTARY MATERIALS FIGURE


