# Supplementary material for: Hypoxia-activated prodrug enhances therapeutic effect of sunitinib in melanoma
Source: Oncotarget. 2017 Dec 5;8(70):115140–52. doi: 10.18632/oncotarget.22944 (PMC5777760; doi:10.18632/oncotarget.22944)
Supplement: Supplementary file 1 [file oncotarget-08-115140-s001.pdf]

Hypoxia-activated prodrug enhances therapeutic effect of sunitinib in melanoma

SUPPLEMENTARY MATERIAL

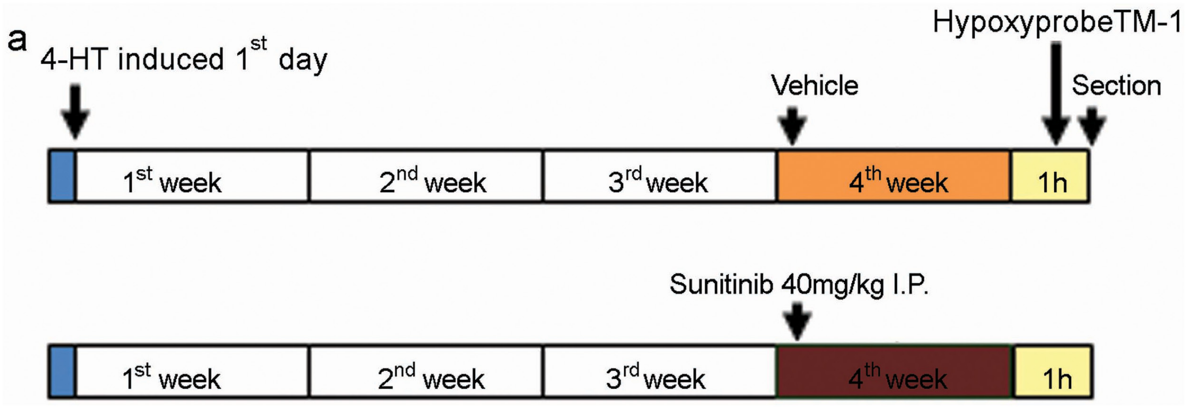

Supplementary Figure 1: Short-term sunitinib increases tumor hypoxia in the GEM melanoma model. Melanomas were induced and allowed to grow for 3 weeks until they were palpable. These mice were then treated for one week with either vehicle control or sunitinib (40mg/kg I.P.) after which they were sacrificed. Tumor tissues were then snap frozen for further analysis.
